# Monkeypox Entry and Emergence Preparation in Pakistan

**DOI:** 10.3390/life13122308

**Published:** 2023-12-08

**Authors:** Saadullah Khattak, Yasir Ali, Zhiguang Ren, Xin-Ying Ji

**Affiliations:** 1 Henan International Joint Laboratory for Nuclear Protein Regulation, School of Basic Medical Sciences, Henan University, Kaifeng 475004, China; saadullah271@gmail.com; 2 National Center for Bioinformatics, Quaid-e-Azam University, Islamabad 45320, Pakistan; yasirkhanqu@gmail.com

## Respected Editor

Monkeypox (Mpox) is a virus that first emerged in Africa in 1970. Belonging to the genus Orthopoxvirus in the family Poxviridae [1], it is spread by contact with animals, people, and infected object [2]. Mpox enters the body via ripped skin and the respiratory tract. A bite, scratch, direct touch with body fluids or lesions, or indirect contact with a lesion all result in animal-to-human transfer [2]. Large respiratory droplets are the means via which most human-to-human transmission occurs, relying on prolonged face-to-face contact, while alternative transmission mechanisms also exist [3]. After the spread of the virus, Infection starts with lethargy, fever, chills, and body pains, similar to smallpox [4]. The infection generates lymphadenopathy, distinguishing Mpox from smallpox and lesions [4]. In recent years, rashes mainly occurred in the urogenital area, although they may occur elsewhere across the body [4]. Mpox has a 1–2-week incubation period and causes a 2–4-week sickness [4,5]. The symptoms and rash may be used to obtained a diagnosis. PCR, enzyme-linked immunosorbent assay, viral isolation, electron microscopy, and immunofluorescent antibody assay are all used confirm results. Once diagnosed, Mpox treatment includes smallpox vaccines (e.g., JYNNEOS^TM^ and ACAM2000) or antiviral drugs such as Tecovirimat, Cidofovir, Vaccinia Immune Globulin Intravenous (VIGIV), and others. JYNNEOS has been approved to prevent Mpox disease spread among people 18 years of age or older and with an elevated susceptibility to Mpox infections. Given the comparable immunological responses to ACAM2000 in both animal research and clinical trials, authorization was granted to this method [6]. However, in extreme instances, the European Medicines Agency has also authorized the use of Tecovirimat [7]. Smallpox vaccinations are only 85% effective, and the WHO reports 167 confirmed fatalities since 1 January 2022 [8]. As a result, stopping viral transmission would be preferable to therapy. Isolation, hand cleanliness, avoiding handling contaminated things and contact with diseased animals are all prevention measures.

The World Health Organization has proclaimed (Mpox) a worldwide health emergency, with over 87,113 confirmed cases as of 24 April 2023 [9]. Although Pakistan has two cases, neighboring countries such as China and India have reported 40 and 22 cases, respectively, with one death due to Mpox as of 24 April 2023 [9]. As of 26 November 2023, the most affected countries globally since 1 January 2022 are United States of America (30,771), Brazil (10,967), Spain (7647), France (4161), Colombia (4090), Mexica (4065), The United Kingdom (3820), Peru (3812), Germany (3757), and China (1935), with over 91,788 cases, 167 deaths, and 116 countries globally reporting Mpox to the WHO. (https://worldhealthorg.shinyapps.io/mpx_global/#2_Global_situation_update, accessed on 11 November 2023). Further occurrences in the WHO Southeast Asia area have been recorded after 18 August 2022, raising the probability of a Mpox epidemic in Pakistan [10,11]. Pakistan, a lower-middle-income nation with a poor healthcare system currently fighting illnesses like polio, must be prepared with a realistic strategy and action plan to deal with the probable Mpox epidemic [10,11]. 

Mpox has recently been detected in nonendemic places, and Mpox-related news has been spreading on social media, putting nations like Pakistan on high alert. The local health service has started to take safeguards, including airport closures. Primary healthcare is open to using resources, yet many projects remain unresolved. Consequently, the system becomes inefficient, and the quality of treatment suffers, further fueling public hatred of the healthcare system. This mistrust reduces people’s desire to seek medical attention and raises the risk of infection [12]. This mistrust originates from a lack of health equality between the affluent and poor as rural regions have inadequate healthcare facilities.

While primary healthcare facilities are present, they are not operational due to a lack of healthcare personnel [12]. Overcrowding at large hospitals is caused by the poor infrastructure of such institutions [12]. The workforce faces challenges due to cultural and linguistic limitations since individuals from various parts of Pakistan have distinct beliefs and dialects [12]. Vaccinations were discontinued in the late 1990s. As a result, the current population’s immunity to Mpox is fading. COVID-19 also affects immune systems by lowering the number of dendritic cells in the body, making patients more susceptible to secondary infections. As a result, even though just two instances of Mpox have been documented in Pakistan since 21 April 2023, Mpox affects the whole urban population and 70% of the rural population.

Pakistan has implemented a national health policy to improve quality of life through physical, social, and mental health, offering numerous preventive health initiatives such as the Expanded Program of Immunization, the National Tuberculosis Control Program, family planning, and school nutrition. Even though such initiatives promise a better future for Pakistanis, the country’s healthcare system is far from ideal. Pakistan has seen many dengue, malaria, and polio endemics, and, most recently, the COVID-19 pandemic [13,14,15]. While polio vaccination is now integrated into the Expanded Program of Immunization (EPI) and disease occurrence has been significantly reduced, the influence the disease still has plays a significant role in illness. These health difficulties have worsened Pakistan’s healthcare capability. A lack of financial resources has made obtaining appropriate human resources, ventilators, hospital beds, and laboratory equipment difficult, making Mpox prevention vital [16]. Pakistan’s population is also at a greater risk of catching Mpox due to a lack of smallpox diagnostic facilities, such as Mpox testing kits. Because Pakistan has a centralized health system, the federal government makes significant health decisions, while the provincial governments implement the plans. However, due to a communication gap and a lack of implementation, duplication outbreak remains a concern because Pakistan lacks the funds, supplies, and infrastructure to control such an outbreak [9]. 

When the WHO declared the Mpox outbreak a global health emergency in July 2022, Pakistan’s Minister for National Health Services (NHS) immediately directed all hospitals and relevant authorities to combat the virus’s spread [14]. Because Pakistan lacks an Mpox diagnostic facility, current policies recommend sending samples abroad for testing if necessary [17]. Physicians’ knowledge of the Mpox virus is critical in limiting the development of an infectious illness in a developing nation like Pakistan. Healthcare personnel should be aware of the presenting signs and symptoms and educated to take a history that allows them to monitor travel history, the source of the infection, and people in close contact. Furthermore, systematically adopting the recommended quarantine and treatment guidelines for Mpox patients would benefit more than just infected patients. It might, however, rule out prospective, asymptomatic, and suspected instances. Government and healthcare officials should make airport screening obligatory and develop national Mpox regulations for the general population [17,18]. Awareness seminars and campaigns should be carefully designed and conducted regularly to educate the public about preventative measures such as social distancing, restricting sexual intercourse, and practicing appropriate hygiene [18]. Educational campaigns that provide factual information regarding the symptoms, treatment, and mild course of Mpox infection might aid in deconstructing widespread misunderstandings [18]. Access to healthcare facilities and screening processes, together with an accurate guide to successfully reporting symptoms and seeking treatment, would empower individuals to tackle the uncertain scenario posed by the Mpox virus. The development of vaccination programs, establishment of screening clinics, and implementation of improved monitoring systems are all essential aims. The ring vaccination technique, which targets susceptible persons who may have been exposed to an infected person at some point, is one of the most successful therapies [18,19].

To summarize, although Pakistan has verified seven cases of Mpox, an epidemic remains a possibility. In Pakistan, a lack of healthcare infrastructure, training, and awareness raises the chance of undiscovered Mpox cases turning into large epidemics. As a result, a concrete action plan must be developed to prepare Pakistan for this possible infrastructural load.
